# Effects of Lignin-Based Hollow Nanoparticle Structure on the Loading and Release Behavior of Doxorubicin

**DOI:** 10.3390/ma12101694

**Published:** 2019-05-24

**Authors:** Yu Zhou, Yanming Han, Gaiyun Li, Fuxiang Chu

**Affiliations:** 1 Research Institute of Wood Industry, Chinese Academy of Forestry, Xiangshan Road, Beijing 100089, China; zhouyu_sky@126.com (Y.Z.); ligy@caf.ac.cn (G.L.); 2 School of Chemistry and Chemical Engineering, Yancheng Institute of Technology, Yancheng 224051, China

**Keywords:** lignin, nanoparticles, vehicles, structure, doxorubicin

## Abstract

Because of their exceptional absorption capacity, biodegradability, and nontoxicity, nanomaterials fabricated from renewable natural resources have recently become an increasingly important research area. However, the mechanism of drug encapsulation by lignin nanoparticles and the role of nanoparticle structure on the stability and loading performance still remain unknown. Herein, lignin hollow nanoparticles (LHNPs) were prepared and applied as promising vehicles for the antineoplastic antibiotic drug doxorubicin hydrochloride (DOX). The hydrogen bonding and π−π interactions contributed to the encapsulation of hydrophilic DOX by LHNPs with hydrophobic cavities. The encapsulation of DOX was enhanced by the pore volume and surface area. In addition, the nanoparticles contributed to the cellular uptake and the accumulation of the drug within HeLa cells. This work provides a scientific basis for future studies on the selective entrapment properties of hollow polymer nanoparticles derived from biomass material as vehicles for overcoming pharmacokinetic limitations.

## 1. Introduction

Lignin is a biomass resource containing a plurality of groups such as methoxy groups, carbonyl groups, carboxyl groups, phenolic hydroxyl groups, and aliphatic hydroxyl groups [1,2]. It is also a potential material for drug delivery because of its properties of biodegradability, biocompatibility, and thermal stability [3,4]. Recently, the output of enzymatic hydrolysis lignin (EHL, a byproduct of biorefinery) has exhibited yearly increases due to the development of the biomass refining industry. Most notably, when amphiphilic lignin molecules are dissolved in a selective solvent, microscopic phase separation occurs with subsequent self-assembly to form particles with a core–shell structure, which provides a simple and feasible path for the high value application of EHL [5]. 

Antineoplastic drugs with antibiotic activity can be highly potent against cancer [6]. However, small molecule drugs can freely diffuse and distribute themselves throughout the body, thereby resulting in undesirable side effects that could limit the achievement of proper doses required to bring about efficacious responses in humans [7,8]. Therefore, it is necessary to use drug delivery systems to protect doxorubicin hydrochloride (DOX) from rapid degradation after systemic distribution [9]. Nanoparticle-based drug delivery carriers have emerged as suitable vehicles for overcoming the pharmacokinetic limitations associated with conventional drug formulations [10].

**Figure 1:**
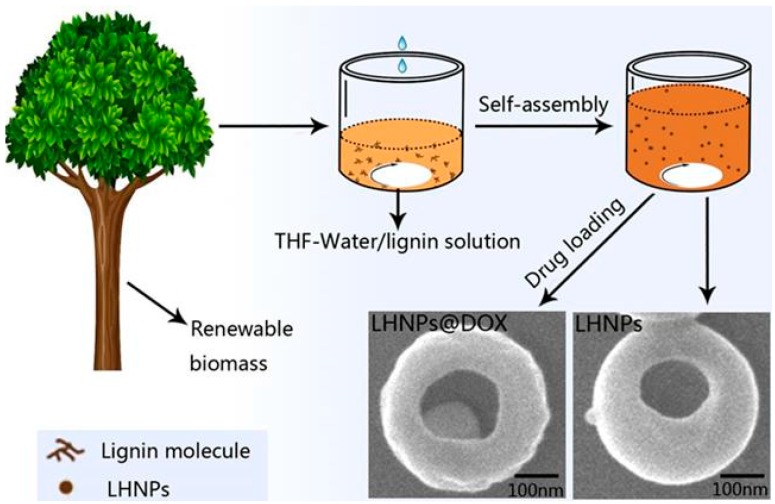
Schematic illustration of the preparation of drug-loaded lignin-based hollow nanoparticles.

Currently, hollow nano- or macroparticles usually show high-quality properties in terms of density, specific surface area, and surface permeability. They are expected to be broadly applied in the medical field [11,12]. DOX is an antibiotic compound with broad antitumor activity, but it has a limited therapeutic index due to toxic side effects that result from an inability of the drug molecules to deeply penetrate into tumor tissues [13,14,15]. Lignin hollow nanoparticles constructed from natural biomass can serve as a high-quality biobased carrier material for DOX (Figure 1).

Our group previously developed lignin hollow nanoparticles via self-assembly [16]. In a previous study, the effects of various processing parameters (predropping lignin concentration, dropping speed of water, and stirring rate) on the structure and size of the lignin hollow nanoparticles were investigated. Currently, several lignin hollow nanoparticles have been prepared [3,17,18,19], but the mechanism of encapsulation and the structural effects on encapsulation of the hollow particles remain unknown. In this study, lignin hollow nanoparticles were prepared using enzymatically hydrolyzable lignin via self-assembly and then were used as a platform to administer DOX. Here, we focused on the mechanism used by the lignin hollow nanoparticles to entrap the drug and the effects of various structures on the encapsulating behavior of the lignin hollow nanoparticles. This work provides a scientific basis for future studies on the selective entrapment properties of hollow polymer nanoparticles.

## 2. Materials and Methods

### 2.1. Materials 

Enzymatically hydrolyzable lignin (EHL) was acquired from Hong Kong Laihe Biotechnology Co., Ltd. The hydroxyl content, the number-average molecular mass, and polydispersity of EHL are 2.67 mmol/g, 1430 g/mol, and 1.22, respectively. Doxorubicin hydrochloride was purchased from Shanghai Macklin Biochemical Co., Ltd. Purity is higher than 98%. THF of analytical grade purity was provided by Beijing Chemical Reagent Company. All other reagents were of analytical grade, and were used as obtained.

### 2.2. Synthesis of Lignin Hollow Nanoparticles

The preparation of lignin hollow nanoparticles (LHNPs) was achieved according to the typical self-assembly method published by Xiong et al. [16] In brief, certain amount of EHL was dissolved in 10 mL tetrahydrofuran (THF) to prepare EHL–THF solution at room temperature, and the solution was stirred with a magnetic stirring bar. Thereafter, 40 mL of water was added dropwise into the solution, which induced the self-assembly of the lignin molecules into colloidal spheres. 

### 2.3. Drug Loading

The DOX-loaded LHNPs were prepared using the coassembly method. In brief, Some DOX was dispersed in THF containing EHL (drug: lignin of 1:10) and stirred for 5 min. Thereafter, 40 mL of water was added dropwise into the solution at a rate of 4 mL/min, which induced the coassembly of the lignin and DOX molecules into colloidal spheres. After 24 h, the colloidal solution was dialyzed against deionized water to remove THF and free compounds. The solution was centrifuged at 11000 rpm for 15 min, and the absorbance of the supernatant was measured at 482 nm with a UV–Vis spectrophotometer (Shimadzu, Kyoto, Japan) to determine the drug concentration. 

The DOX loading and encapsulation efficiency were calculated according to the following equations.

DOX loading (%) = (weight of DOX loaded / weight of LHNPs@DOX) × 100

Encapsulation efficiency (%) = (weight of DOX loaded / weight of initial amount of DOX) × 100

### 2.4. In Vitro Release Studies

The in vitro release of DOX from the LHNPs was investigated using dialysis against PBS (pH 7.4 and 5.5). In brief, 1 mg of pure drug and 10 mg of drug-loaded LHNPs were immersed in 50 mL PBS, and then centrifuged at a speed of 100 rpm and a temperature of 37 °C. At scheduled intervals, 5 mL of medium was collected and replaced with the same amount of fresh release medium. The samples were centrifuged at 11000 rpm for 5 min, and the absorbance (i.e., amount of DOX released) was measured at 482 nm with a UV spectrophotometer (Shimadzu, Kyoto, Japan). The average calculated values were obtained from at least three replicates.

### 2.5. Cellular Uptake of Modified LHNPs Assays

Confocal laser scanning microscopy (CLSM, Olympus Corporation, Tokyo, Japan) was used to study the cellular uptake of the LHNPs. HeLa cells were plated and cultured in six-well plates. Thereafter, the LHNPs were FITC-labeled (labeled by fluorescein isothiocyanate) track the distribution of nanoparticles during the assay. After incubation for 24 h, different concentrations (LHNPs served as a standard) of the modified LHNPs were added into the wells. After incubation for 4 and 24 h, the cells were washed three times with PBS and fixed with paraformaldehyde in PBS. Images of the uptake were obtained by CLSM.

## 3. Results and Discussion

### 3.1. Morphology of EHL, Lignin Hollow Nanoparticles, and Drug-Loaded Lignin Nanoparticles

**Figure 2:**
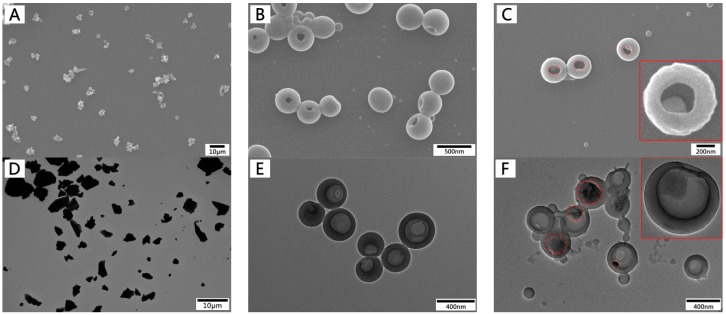
SEM (**A**–**C**) and TEM (**D**–**F**) images of enzymatic hydrolysis lignin (EHL), lignin hollow nanoparticles (LHNPs), and LHNPs@ doxorubicin hydrochloride (DOX) at predropping lignin concentration of 1mg/mL, stirring rate of 600 rmp, and dropping speed of water of 4mL/min.

The lignin raw materials consisted of irregularly shaped block structures with particle sizes ranging from several microns to dozens of microns, as shown in Figure 2A,D. Figure 2B,E shows how the lignin nanoparticles were morphologically constructed by EHL via self-assembly under the conditions of an initial lignin concentration of 1 mg/mL, a water dropping speed of 4 mL/min, and a stirring rate of 600 rpm. A scanning electron microscopy (SEM) image of lignin nanoparticles shows the spherical hollow structure with a single hole on the surface of the particles. A transmission electron microscopy (TEM) image displays a clear contrast between the center and the shell, which supplies further evidence that the particles are hollow. The nanoparticle structure did not significantly change after loading the drug. However, a bulk structural material was found in the cavity, as shown in Figure 2C,F. It is likely that the bulk structure in the cavity was composed of crystals of DOX after drying (TEM images and the X-ray diffraction (XRD) pattern of the DOX crystals are shown in Figure S1). 

**Figure 3:**
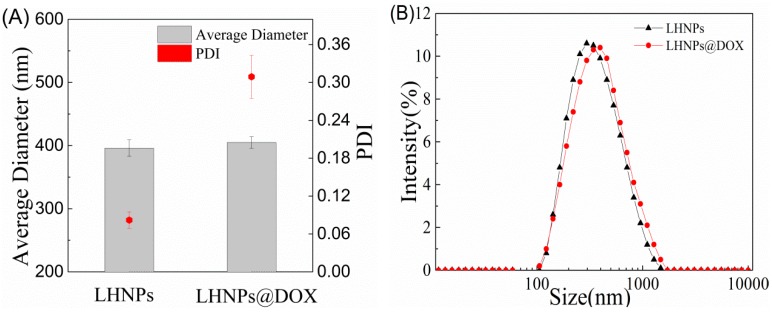
Characterization of LHNPs and DOX-loaded LHNPs. (**A**) Size and polydispersity index (PDI); (**B**) The size distribution by dynamic light scattering (DLS).

In Figure 3, the dynamic light scattering (DLS) measurements revealed that the particles exhibited a narrow size distribution, and the average sizes of the LHNPs and DOX-loaded LHNPs were 396 ± 13 and 405 ± 9 nm, respectively.

In Figure 3, the dynamic light scattering measurements revealed that the particles exhibited a narrow size distribution and the average sizes of the LHNPs and DOX-loaded LHNPs were 396 ± 13 and 405 ± 9 nm, respectively.

### 3.2. Morphological Structure Regulation of Lignin Hollow Nanoparticles

**Figure 4:**
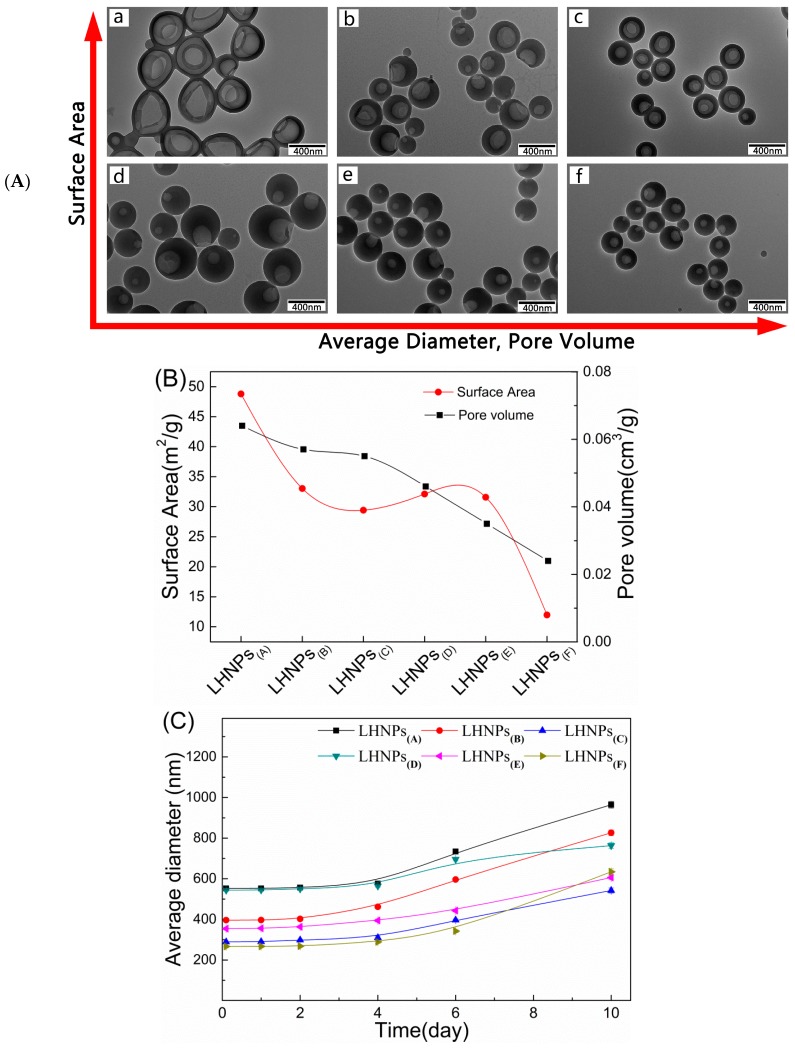

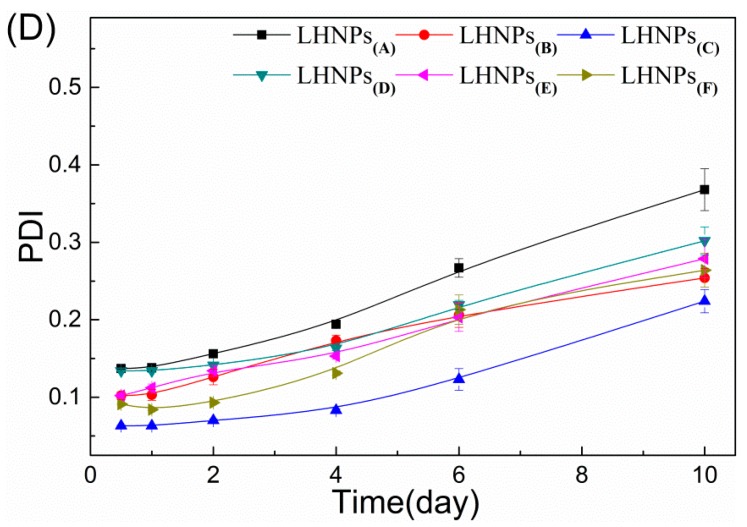
(**A**) TEM images of LHNPs at different preparation conditions; (**B**) Surface area and pore volume of the nanoparticles. Stability of LHNPs after 10h incubation in PBS (pH 7.4) at 37 °C, effects on the average diameter (**C**); PDI (**D**).

Because the lignin hollow nanoparticle structure has significant effects on the encapsulation and the sustained release of the drug, the morphological structure of LHNPs regulated by predropping lignin concentration, dropping speed of water, and stirring rate was studied. Figure 4 shows the TEM image of a series of LHNPs prepared under different conditions (the process parameters are shown in Table S1). The values of surface area and pore volume of the LHNPs are exhibited in Figure 4B. Through a comparison of the morphological structure of the LHNPs on the transverse and vertical axes, the growth laws of micro-nanospheres were preliminarily obtained. The shell thickness and predropping lignin concentration are proportional to each other, and the concentration determined the shell thickness by controlling the amount of molecules participating in the formation of the particle shell in the initial stage of self-assembly [20]. The self-assembly time of lignin was affected by the speed of water as it dropped [4]. The faster the drop acceleration, the smaller the pore volume. Moreover, an increase in the stirring rate could lead to an increase in the shear force, resulting in a decrease in the prepared nanoparticle size [21]. The above results indicated that the LHNPs were a morphological structure-controlled polymer hollow nanoparticle.

The stability of the LHNPs was evaluated at a physiological pH by incubating the LHNPs with phosphate-buffered saline (PBS, pH 7.4) at 37 °C for 10 days. The LHNPs maintained a constant size for 5 days in PBS (Figure 4C), which was indicative of a high colloidal stability without aggregate formation. In addition, the polydispersity index (PDI) values of the nanoparticles were nearly unchanged during this time (Figure 4D). However, the long-term immersion of the nanoparticles in PBS and the diameter of the single hole of the particles affected the electrical double layer structure, thereby causing particle aggregation. Thus, we observed an increase in the average diameter and the PDI after 5 days.

### 3.3. Drug Loading and In Vitro Release Studies

**Figure 5:**
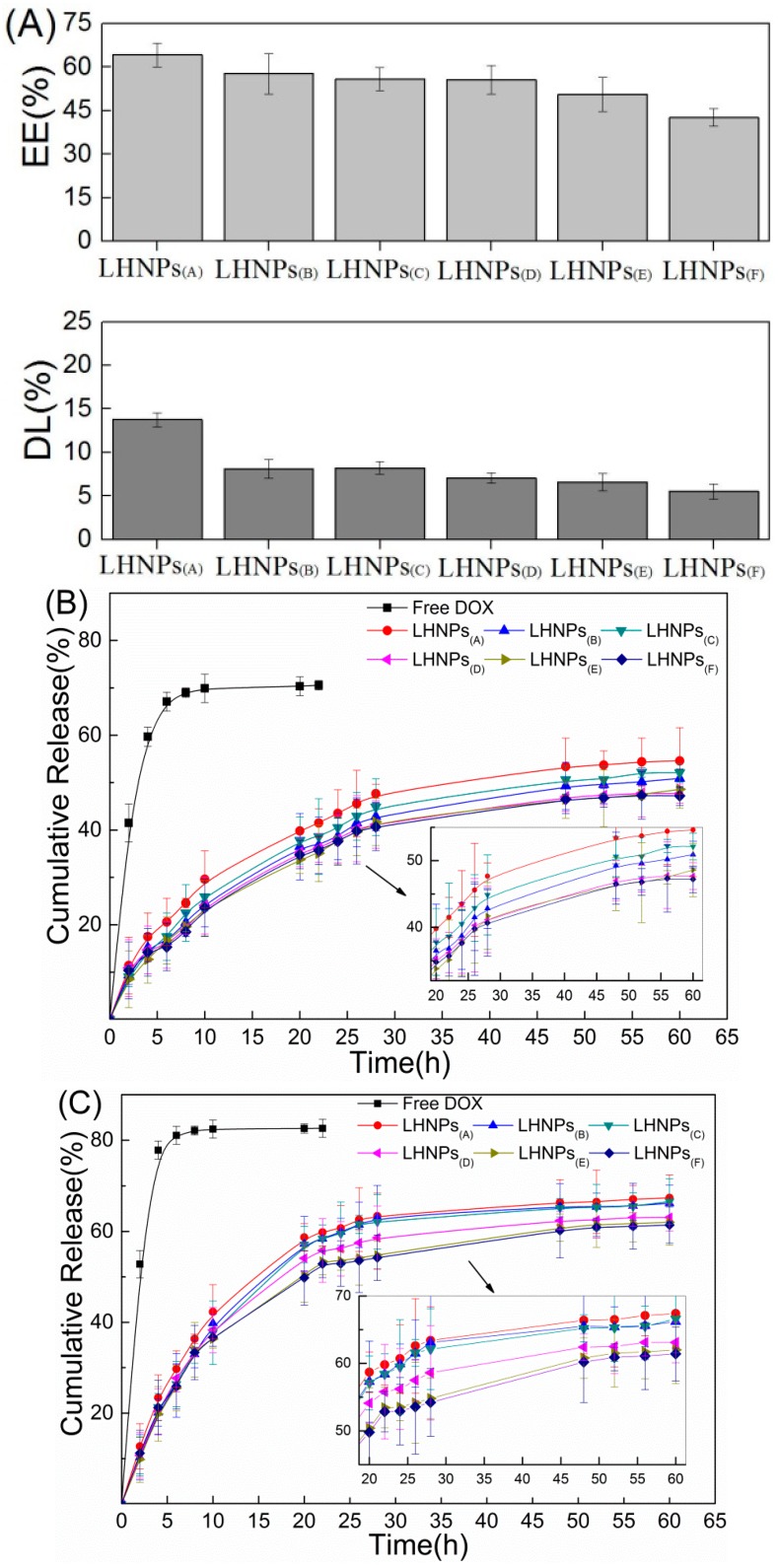
(**A**) Drug loading (EL) and encapsulation efficiency (EE) of the LHNPs; (**B**) Release profiles of DOX from DOX@LHNPs at pH 5.5; (**C**) Release profiles of DOX from DOX@ LHNPs at pH 7.4.

The values for drug loading (DL) and encapsulation efficiency (EE) of the LHNPs are shown in Figure 5A. Compared with Figure 4B, it can be seen that the encapsulation efficiency and drug loading of the nanoparticles are related to the surface area and pore volume. 

The drug release profile of LHNPs@DOX was evaluated in two different aqueous solutions in order to simulate the tumor microenvironment (pH 5.5) and physiological pH conditions (pH 7.4), and the results are shown in Figure 5B,C. The results clearly revealed a difference in DOX release between the two pH systems. For the same test object, the drug released rapidly in acidic (pH 5.5) conditions compared with neutral (pH 7.4) conditions. In addition, DOX@LHNPs(A), DOX@LHNPs(B), and DOX@LHNPs(C) released the drug faster than the other particles exposed to the two pH systems, which could have been attributed to the larger surface area and wider pore volume. Furthermore, the figure revealed that DOX@LHNPs(D), DOX@LHNPs(E), and DOX@LHNPs(F) exhibited more stable cumulative release than the other nanoparticles. This was attributed to their thicker shell and smaller pore size, which could better protect the nanoparticles against the swelling effect caused by PBS [22]. Due to the effects of the shell, the DOX-loaded nanoparticles exhibited a sustained release process because the loaded DOX had a stable storage site, as shown in the drug release profile [23].

These results indicate that the difference between the loading and the releasing performance of DOX can be due to the different structures, sizes, and stabilities of these LHNPs. The shell thickness of the polymer particle plays a vital role in capsule-based slow or controlled release applications [24,25]. The controlled release behavior of the LHNPs, the entry of DOX@LHNPs into cells, and the acidic environment of lysosomes can facilitate the release of DOX to achieve more powerful therapeutic effects [26]. Overall, the above results provide new insights into the selective encapsulation of LHNPs that can be used in other fields by investigating the effects of different lignin nanoparticle structures on the DOX-loading behavior.

### 3.4. Formation Mechanism of DOX-Loaded Lignin Nanoparticles

**Figure 6:**
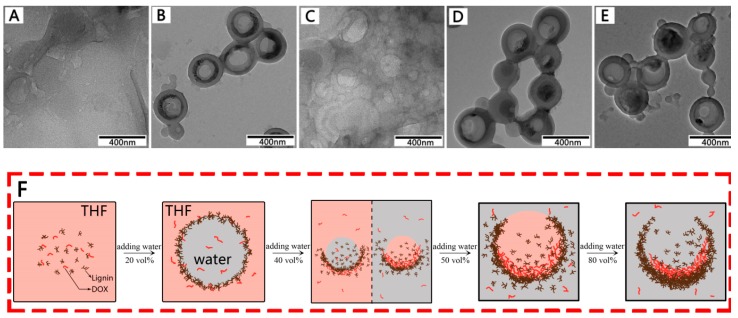
TEM images of the samples obtained from the dispersions at different water contents. The predropping lignin concentration was 1mg/mL, stirring rate of 600 rpm, and dropping speed of 4 mL/min. (**A**) 0 vol %, (**B**) 20 vol %, (**C**) 40 vol %, (**D**) 50 vol %, (**E**) ≥80 vol %, and (**F**) schematic representation of formation process of the drug-loaded lignin hollow nanoparticles in THF-H_2_O.

Previous studies reported that analytical reagent grade tetrahydrofuran (AR-THF) contains impurities (toluene, trimethylphosphine oxide, butylated hydroxytoluene, and triethyl citrate) that lead to phase separation between tetrahydrafuran (THF) and water. Furthermore, the phase separation forms a nanoemulsive substance [16,27]. The process of formation of the drug-loaded lignin hollow nanoparticles is shown in Figure 6F. EHL and DOX were dissolved in THF to form a mixed solution, and no particles could be seen on the TEM images (Figure 6A). Phase separation, followed by the continuous phase (THF) and the dispersed phase (water), occurred when deionized water was added dropwise into the mixed solution. The lignin nanoparticles took shape as the hydrophobic parts of EHL formed a membrane layer to wrap the water at the two-phase interface (Figure 6B) [28]. At this point, because the polarity of deionized water is much higher than that of THF and the solubility of DOX in water is greater than that of THF, DOX entered into the water phase through the lignin membrane (the concentration of DOX in water was 10 mg/mL, and the concentration in THF was less than 0.7 mg/mL). 

In order to reduce the pressure between the inside and outside of the shell, phase inversion occurred, and the particles recombined when the water content was 40 vol% (Figure 6C). The electrostatic forces and π−π interactions could cause the DOX to adhere to the inner wall of the membrane layer [29]. Therefore, the drug was still encapsulated in the cavity after particle reorganization. When the water content was 50 vol%, a single hole appeared at the weak part of the particle shell (Figure 6D) [30]. The drug-loaded lignin hollow nanoparticles were completely formed at a water content of 80 vol%. The final drug-loaded nanoparticles are shown in Figure 6E.

**Figure 7:**
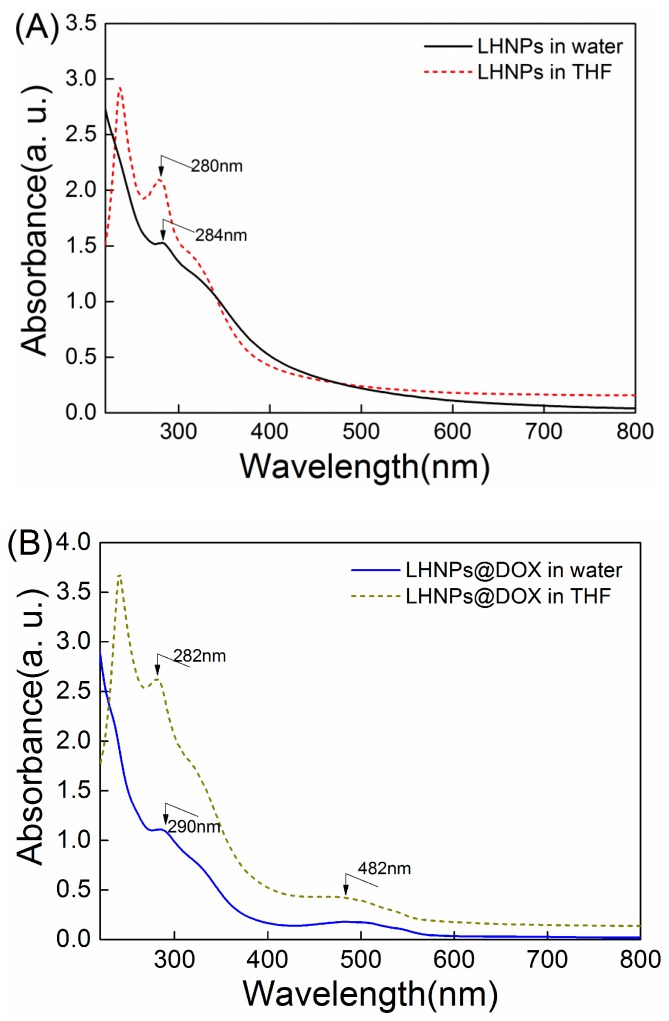
UV–Vis absorption spectra of (**A**) LHNPs and (**B**) LHNPs@DOX.

The efficiency of DOX encapsulation was 61.5±6%. However, the encapsulation efficiency when adding DOX to a colloidal LHNP solution to form drug-loaded LHNPs was 50.1±7%, with strong interactions between the carrier material and the drug molecules [7]. In addition to the adsorption action of DOX, π−π stacking also enhanced the interactions between DOX and EHL, as the polyphenolic structure of the lignin molecules is similar to that of DOX. These results were confirmed by UV–Vis absorption spectra (Figure 7). As shown in Figure 7A, the absorption peak of the lignin hollow nanoparticles in water was 284 nm, whereas the red-shifted absorption peak returned to 280 nm after redissolving in THF. 

These results demonstrated the existence of π−π interactions between the lignin molecules during the preparation of LHNPs [16]. Furthermore, absorption peaks corresponding to DOX-loaded nanoparticles were observed at 290 nm and 282 nm for the nanoparticles re-dissolved in deionized water and THF, respectively (Figure 7B). The red-shifted absorption peaks corresponding to the two types of nanoparticles in water were different from those after redissolving in THF, which confirmed the existence of π−π interactions between the lignin and DOX molecules in the assembly of DOX-loaded LHNPs. The Fourier-transform infrared (FTIR) spectrum showed no disappearance of chemical bonds and no appearance of new absorption peaks, except for the characteristic peaks (1622 cm^-1^, 1590 cm^-1^) of DOX compared to the pure LHNPs (Figure S2). These results demonstrated that electrostatic adsorption and π−π interactions resulted in DOX being encapsulated within LHNPs.

### 3.5. LHNPs and DOX-Loading LHNPs Cellular Uptake

**Figure 8:**
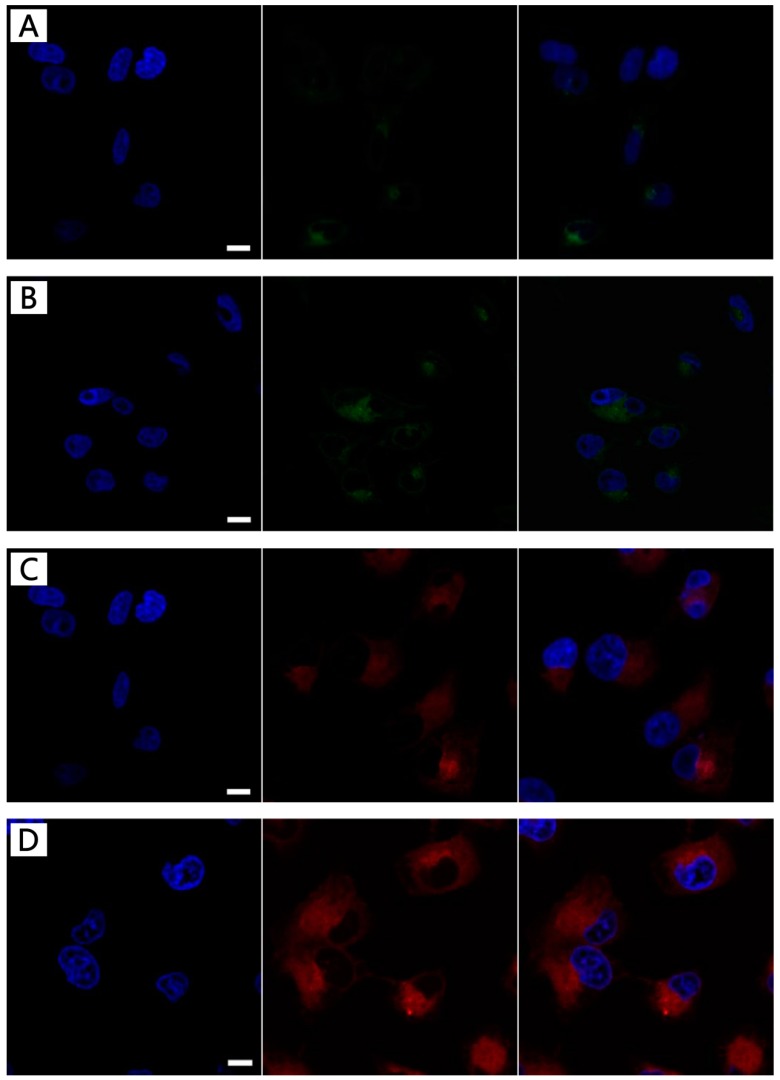
The localization of FITC-modified LHNPs and DOX-loaded LHNPs from confocal laser scanning microscopy (CLSM): FITC–LHNPs (**A** and **B**), DOX-loaded LHNPs in HeLa cells for 4h (A, **C**) and 12 h (B, **D**). Bar: 1μm.

To investigate the cellular uptake and intracellular drug release of drug-loaded lignin nanoparticles, a live cell imaging system was used. As shown in Figure 8, it was observed that the fluorescence intensity in the HeLa cell became stronger with an increase in the incubation time, indicating that a long incubation time was beneficial for cellular uptake in these cells. Thereafter, the cellular uptake of DOX-loading nanoparticles was examined by CLSM to test the uptake effect by comparing the red fluorescence intensity in HeLa cells after incubation for 4 and 24 h (Figure 8D). A red fluorescent signal was noted in the cytoplasm after incubation for 4 h and, after 24 h, partial blue fluorescence overlapped with the red fluorescence, indicating that the drug was delivered into the nucleus to inhibit the proliferation of HeLa cells. This phenomenon could be attributed to the acidic environment inside the tumor cells that induced the disintegration of the nanostructures after a long period of time [31]. Free DOX entered into cells by diffusion, and the drug could freely escape from the cells. However, it was not easy for the drug-loaded nanoparticles to escape from the cells once they were internalized [10,32,33]. These results were consistent with those from the cellular uptake of the unloaded nanoparticles.

## 4. Conclusions

In summary, we encapsulated DOX within LHNPs, explored the mechanism of encapsulation, and studied the effects of the nanoparticle structure on the encapsulation behavior of DOX. Due to the synergistic effects between the adsorption force and π−π interactions, the DOX molecules were firmly encapsulated within the cavity of the LHNPs during the assembly of lignin and DOX. The structural effects of the lignin nanoparticles on the stability and loading performance were also investigated during DOX preparation, encapsulation, and release. These sustainable lignin nanoparticles with a spherical hollow structure could be used as potential vehicles for compounds with benzene rings due to their exceptional absorption capacity, biodegradability, and nontoxicity.

## Supplementary Materials

The following are available online at https://www.mdpi.com/1996-1944/12/10/1694/s1, Figure S1: TEM images of crystallization DOX and XRD pattern of DOX after drying, Figure S2: FTIR spectra of LHNPs and DOX-loaded LHNPs, Table S1: Process parameters of the LHNPs.

## References

[1] Duval A., Lawoko M. (2014). A review on lignin-based polymeric, micro- and nano-structured materials. React. Funct. Polym..

[2] Laurichesse S., Avérous L. (2014). Chemical modification of lignins: Towards biobased polymers. Prog. Poly. Sci..

[3] Figueiredo P., Lintinen K., Kiriazis A., Hynninen V., Liu Z., Baulethramos T., Rahikkala A., Correia A., Kohout T., Sarmento B. (2017). In vitro evaluation of biodegradable lignin-based nanoparticles for drug delivery and enhanced antiproliferation effect in cancer cells. Biomaterials.

[4] Li H., Deng Y., Liu B., Ren Y., Liang J., Qian Y., Qiu X., Li C., Zheng D. (2016). Preparation of nanocapsules via the self-assembly of kraft lignin: A totally green process with renewable resources. ACS Sustain. Chem. Eng..

[5] Hong N., Li Y., Qiu X. (2016). A highly efficient dispersant from black liquor for carbendazim suspension concentrate: Preparation, self-assembly behavior and investigation of dispersion mechanism. J. Appl. Polym. Sci..

[6] Flavahan W.A., Gaskell E., Bernstein B.E. (2017). Epigenetic plasticity and the hallmarks of cancer. Science.

[7] Blanco E., Shen H., Ferrari M. (2015). Principles of nanoparticle design for overcoming biological barriers to drug delivery. Nat. Biotechnol..

[8] Mou X., Ali Z., Li S., He N. (2015). Applications of magnetic nanoparticles in targeted drug delivery system. J. Nanosci. Nanotechnol..

[9] Chidambaram M., Manavalan R., Kathiresan K. (2011). Nanotherapeutics to overcome conventional cancer chemotherapy limitations. J. Pharm. Pharm. Sci..

[10] Qiu M., Sun H., Meng F., Cheng R., Zhang J., Deng C., Zhong Z. (2018). Lipopepsomes: A novel and robust family of nano-vesicles capable of highly efficient encapsulation and tumor-targeted delivery of doxorubicin hydrochloride in vivo. J. Control. Release.

[11] Si Y., Chen M., Wu L. (2016). Syntheses and biomedical applications of hollow micro-/nano-spheres with large-through-holes. Chem. Soc. Rev..

[12] Xu J., Ma A., Xu Z., Liu X., Chu D., Xu H. (2016). Synthesis of au and pt hollow capsules with single holes via pickering emulsion strategy. J. Phys. Chem. C.

[13] Carvalho C., Santos R.X., Cardoso S., Correia S., Oliveira P.J., Santos M.S., Moreira P.I. (2009). Doxorubicin: The good, the bad and the ugly effect. Curr. Med. Chem..

[14] Minotti G., Menna P., Salvatorelli E., Cairo G., Gianni L. (2004). Anthracyclines: Molecular advances and pharmacologic developments in antitumor activity and cardiotoxicity. Pharmacol. Rev..

[15] Mcrae P.S., Henchey E., Chen X., Schneider S., Emrick T. (2014). Efficacy of polympc-dox prodrugs in 4t1 tumor-bearing mice. Mol. Pharm..

[16] Xiong F., Han Y., Wang S., Li G., Qin T., Chen Y., Chu F. (2017). Preparation and formation mechanism of renewable lignin hollow nanospheres with a single hole by self-assembly. ACS Sustain. Chem. Eng..

[17] Chen N., Dempere L.A., Tong Z. (2016). Synthesis of pH-responsive lignin based nanocapsules for controlled release of hydrophobic molecules. ACS Sustain. Chem. Eng..

[18] Dai L., Liu R., Hu L., Zou Z., Si C. (2017). Lignin nanoparticle as a novel green carrier for the efficient delivery of resveratrol. ACS Sustain. Chem. Eng..

[19] Li Y., Zhou M., Pang Y., Qiu X. (2017). Lignin-based microsphere: Preparation and performance on encapsulating the pesticide avermectin. ACS Sustain. Chem. Eng..

[20] Lievonen M., Valle-Delgado J.J., Mattinen M.L., Hult E.L., Lintinen K., Kostiainen M.A., Paananen A., Szilvay G.R., Setälä H., Österberg M. (2016). Simple process for lignin nanoparticle preparation. Green Chem..

[21] Mateovic T., Kriznar B., Bogataj M., Mrhar A. (2002). The influence of stirring rate on biopharmaceutical properties of eudragit rs microspheres. J. Microencapsul..

[22] Liu Z., Qie R., Li W., Hong N., Li Y., Li C., Wang R., Shi Y., Guo X., Jia X. (2017). Preparation of avermectin microcapsules with anti-photodegradation and slow-release by the assembly of lignin derivatives. New J. Chem..

[23] Liu J., Pang Y., Huang W., Zhu Z., Zhu X., Zhou Y., Yan D. (2011). Redox-responsive polyphosphate nanosized assemblies: A smart drug delivery platform for cancer therapy. Biomacromolecules.

[24] Sheng W.B., Li W., Zhang G.X., Tong Y.B., Liu Z.Y., Jia X. (2015). Study on the uv-shielding and controlled-release properties of a polydopamine coating for avermectin. New J. Chem..

[25] Antipov A.A., Sukhorukov G.B., Edwin Donath A., Möhwald H. (2001). Sustained release properties of polyelectrolyte multilayer capsules. J. Phys. Chem. B.

[26] Yu P., Xia X.M., Wu M., Cui C., Zhang Y., Liu L., Wu B., Wang C.X., Zhang L.J., Zhou X. (2014). Folic acid-conjugated iron oxide porous nanorods loaded with doxorubicin for targeted drug delivery. Colloids Surf. B Biointerfaces.

[27] Xiong F., Chu F., Li G., Wang S., Qin T., Han Y., Chen Y. (2017). Preparation and formation mechanism of size-controlled lignin nanospheres by self-assembly. Ind. Crop. Prod..

[28] Norato M.A., Tavlarides L.L., Tsouris C. (2010). Phase inversion studies in liquid-liquid dispersions. Can. J. Chem. Eng..

[29] Deng Y., Zhao H., Qian Y., Lei L., Wang B., Qiu X. (2016). Hollow lignin azo colloids encapsulated avermectin with high anti-photolysis and controlled release performance. Ind. Crop. Prod..

[30] Lv H., Lin Q., Zhang K., Yu K., Yao T., Zhang X., Zhang J., Yang B. (2008). Facile fabrication of monodisperse polymer hollow spheres. Langmuir ACS J. Surf. Colloids.

[31] Ding J., Liang T., Zhou Y., He Z., Min Q., Jiang L., Zhu J. (2017). Hyaluronidase-triggered anticancer drug and sirna delivery from cascaded targeting nanoparticles for drug-resistant breast cancer therapy. Nano Res..

[32] Wang H., He J., Zhang M., Tao Y., Li F., Tam K.C., Ni P. (2013). Biocompatible and acid-cleavable poly(ε-caprolactone)-acetal-poly(ethylene glycol)-acetal-poly(ε-caprolactone) triblock copolymers: Synthesis, characterization and ph-triggered doxorubicin delivery. J. Mater. Chem. B.

[33] Dai L., Zhu W., Liu R., Si C. (2018). Lignin-containing self-nanoemulsifying drug delivery system for enhance stability and oral absorption of trans-resveratrol. Part. Part. Syst. Charact..

