# Supplementary material for: Effects of Lignin-Based Hollow Nanoparticle Structure on the Loading and Release Behavior of Doxorubicin
Source: Materials (Basel). 2019 May 24;12(10):1694. doi: 10.3390/ma12101694 (PMC6566404; doi:10.3390/ma12101694)
Supplement: Supplementary file 1 [file materials-12-01694-s001.pdf]

Supplementary Information

Effects of Lignin-Based Hollow Nanoparticle Structure on the Loading and Release Behavior of Doxorubicin

Yu Zhou[1,2], Yanming Han [1,*], Gaiyun Li [1] and Fuxiang Chu [1,*]

[1] Research Institute of Wood Industry, Chinese Academy of Forestry, Xiangshan Road, Beijing 100089, China

[2] School of Chemistry and Chemical Engineering, Yancheng Institute of Technology, Yancheng 224051, China

* Correspondence: hanym@caf.ac.cn (Y.H.); chufuxiang@caf.ac.cn (F.C.); Tel./Fax: +86-10-62889433

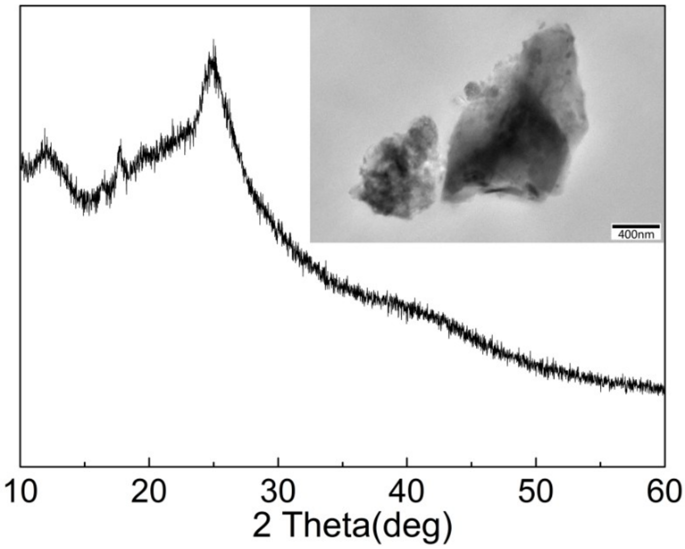

Figure S1. TEM images of crystallization DOX and XRD pattern of DOX after drying.

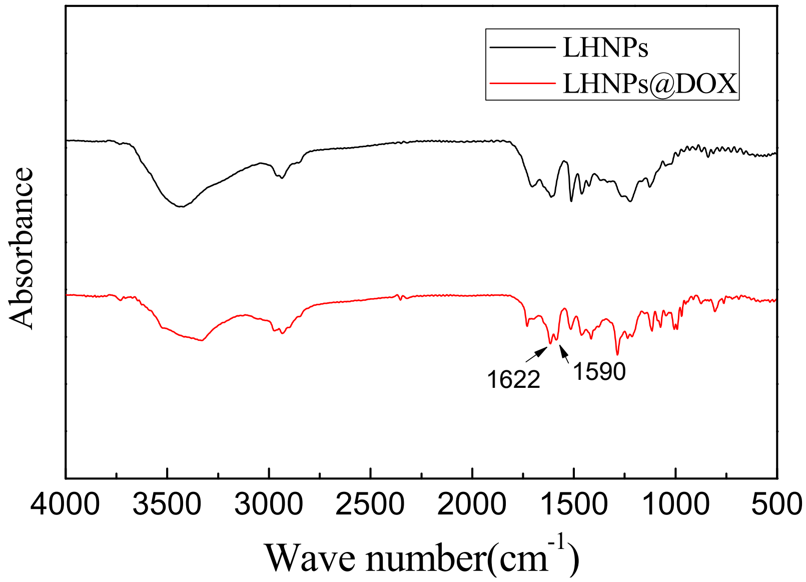

Figure S2. FTIR spectra of LHNPs and DOX-loaded LHNPs

Table S1. Process parameters of the LHNPs.

Nanoparticles	Pre-dropping Lignin Concentration (mg/mL)	Dropping Speed of Water (mg/min)	Stirring Rate (rpm)
$LHNPs_{(A)}$	0.3	4	700
$LHNPs_{(B)}$	1	4	200
$LHNPs_{(C)}$	1	7	700
$LHNPs_{(D)}$	1	2	700
$LHNPs_{(E)}$	3	4	1200
$LHNPs_{(F)}$	3	4	700
